# Introduction to ‘Medicinal Chemistry Small Molecule Probes’

**DOI:** 10.1039/d4cb90005g

**Published:** 2024-03-25

**Authors:** Gemma Nixon, Khondaker Miraz Rahman, John Spencer

**Affiliations:** a Department of Chemistry, University of Liverpool Crown Street Liverpool L69 7ZD UK; b Institute of Pharmaceutical Science, King’s College London 150 Stamford Street London SE1 9NH UK; c Sussex Drug Discovery Centre, School of Life Sciences, University of Sussex Falmer BN1 9QJ UK j.spencer@sussex.ac.uk

## Abstract

Gemma Nixon, Khondaker Miraz Rahman and John Spencer introduce the *RSC Chemical Biology* themed collection on ‘Medicinal Chemistry Small Molecule Probes’.
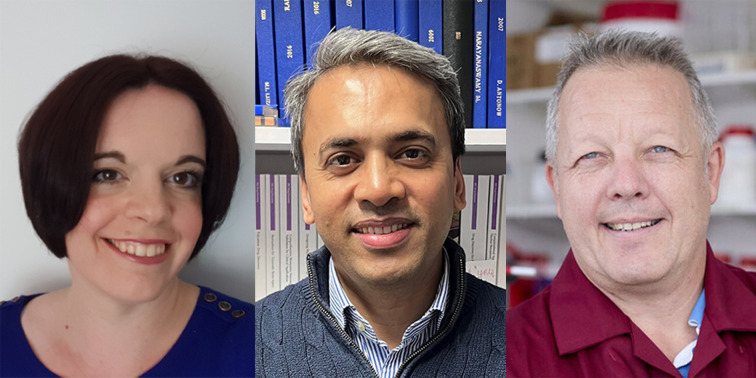

We are delighted to welcome you to this innovative and exciting themed collection, which features contributions in the fields of contemporary medicinal chemistry and chemical biology. These span a range of topics, including small molecule drugs, diagnostics, antiviral peptides and macrocyclic libraries.

In their opinion article (https://doi.org/10.1039/D3CB00082F), Spencer and colleagues present case studies of medicinal chemistry projects where provision of the wrong stereoisomer has led to discrepancies in research. For example, the wrong enantiomer, *e.g. R*- instead of *S*-, is unlikely to bind to its target protein and may result in toxicity. Be vigilant, ask for full characterisation and purchase materials from reputable suppliers, are key take-home messages.

Sialic acids are sugar derivatives with numerous roles in multiple diseases, including cell surface phenomena implicated in influenza, parasitic and coronavirus infections. Field and coworkers (https://doi.org/10.1039/D3CB00155E) provide an extensive overview of the structural, mechanistic and drug discovery aspects of this exciting family of natural products.

Temozolomide, often combined with radiation, is a clinically used drug for various brain cancers, termed gliomas. In their review (https://doi.org/10.1039/D3CB00076A) Stevens and Wheelhouse highlight the pioneering work, much of which emanates from their UK-based laboratory. They shed light on the prodrug action of this interesting family of imidazotetrazines. They also raise the concerns of limited funding and slow progress, since their initial discoveries some four decades ago, for a range of diseases that need improved treatment options.

Hodgkinson and coworkers (https://doi.org/10.1039/D3CB00105A) provide an overview of the important field of epigenetic modulators, specifically HDAC (histone deacetylase) inhibitors. These compounds inhibit the post-translational modification (PTM) that leads to the removal of acetyl groups on acetylated lysine residues in proteins. The interplay between acetylation (neutral state) and deacetylation (charged state) influences gene transcription in cells. However, the large number of HDAC enzymes complicates biology and isoform-specific inhibitors are necessary. Noticeable improvements in past years have included the development of heterobifunctional molecules such as proteolysis targeting chimeras (PROTACs). This class of molecules promote degradation of a protein of interest by hijacking the ubiquitin-proteasome process in cells. Despite appearing to be non-“Lipinski” compliant, these modalities are enjoying success in the clinic. It is anticipated that HDAC-targeting PROTACs are just around the corner and would represent a significant advancement of the field. This will be a major achievement for the authors, adding to their recent success in the Great British Bake Off, and benefit others working in this area.

Brimble and colleagues review the design of peptide-based antivirals, notably the development of P_1_ glutamine isosteres targeting 3C/3CL^pro^ (https://doi.org/10.1039/D3CB00075C). Since viral infections are amongst the leading causes of human morbidity, and with the recent covid pandemic, their review is particularly timely. Meanwhile, in their communication, Miller and colleagues, using a combination of virtual and in-vitro screening, identified an unprecedented small-molecule covalent inhibitor of guanosine diphosphate mannose dehydrogenase with an IC_50_ value in the high micromolar range (https://doi.org/10.1039/D3CB00126A). This could be potentially very impactful in the cystic fibrosis area, where *Pseudomonas aeruginosa*-derived bacterial infections are rife.

O’Neill and colleagues, in their paper (https://doi.org/10.1039/D3CB00109A), describe the synthesis of photoreactive chemical probes for the proteomic study of antimalarials. These probes, with their novel modes of action, could be vital tools in the battle against malaria resistance, which has resulted from the extensive deployment of artemisinin combination therapies (ACTs). Xu and Wagner describe a cell-permeable and stable pyrazol-3-one-based probe label of LgtC, a bacterial glycosyltransferase and virulence factor, which has uses in the detection of bacterial strains (https://doi.org/10.1039/D3CB00092C). This will help plug the gap in such probes, which tend to be charged, and hence, poorly cell permeable.

A photoswitch for peptide stapling is described by Spring and colleagues (https://doi.org/10.1039/D3CB00176H). This works on an S_N_Ar process involving a cysteine residue and avoids the UV damage and poor skin penetration of UV light, employed in many other similar switches.

The field of covalent drugs is experiencing a resurgence, with several clinical successes. Barker and colleagues describe an ATP-binding-site lysine- and tyrosine-residue-targeting fluorosulfate covalent inhibitor of P14iIIIB, a lipid kinase (https://doi.org/10.1039/D3CB00142C). High selectivity is achieved due to its initial reversible inhibition prior to its covalent action. Schofield and colleagues describe a mass-spectrometry-based assay on Jumonji-C (JmjC)-domain-containing protein 5 (JMJD5), a human 2-oxoglutarate (2OG) iron-dependent oxygenase (https://doi.org/10.1039/D2CB00249C). This assay facilitated the identification of selective JMJD5 inhibitors. Finally, Mondal and co-workers, through comprehensive proteomics and target engagement studies in cells, describe cyanopyrolidine warhead-containing activity-based probes for the deubiquitinating enzyme USP30.

The collection highlights the great strides made in drug discovery and development over the last few decades, showcasing a broad variety of research. These achievements have notably been made possible through improved chemical-library generation and better knowledge of structural biology, ultrasensitive biophysical measurements, target identification and genetics. We hope that you find this to be a very useful collection of contemporary medicinal chemistry and chemical biology.

## Supplementary Material

